# War Surgery of the Chest

**Published:** 1918

**Authors:** 


### SURGERY

**War Surgery of the Chest.** By A. L. Lockwood, Captain, R. A. M. C., and J. A. Nixon, Captain, R. A. M. C. Abs. from the *Brit. Med. Jour.*, Jan. 26 and Feb. 2, 1918.

The authors recall that until about the middle of July, 1916, it was generally not thought necessary to excise parietal wounds unless they were badly infected. At that time it was realized that all wounds of the diaphragm left unrepaired proved fatal, and that simple repair was not always sufficient.

Chest cases that require surgical treatment — especially traumatopnea — require it as early and as promptly as abdominal cases. Suture at the advanced dressing station or even the regimental aid post is desirable.

Such cases are put in warm blankets at the casualty clearing station. Continuous rectal administration of soda bicarbonate is given if required. In severe cases blood transfusion is indicated. Hot drinks may be taken but no stimulants. Every effort should be made to induce sleep and, if necessary, omnopon or morphine may be administered. Dyspnea from hemothorax or pneumothorax should be relieved before operation by aspirating. Serious cases should be operated as soon as conditions permit.

Careful localization of the missile by X-ray and repeated X-ray examinations during the progress of the case are essential. If the missile is observed to have traversed the mediastinum and penetrated both sides of the chest, early operative treatment should never be undertaken.

A separate theater is reserved for chest surgery and a uniform temperature of 80° is maintained. One ampoule of omnopon-scopolamine is administered an hour before the operation. If not sleeping, the patient is given half of an ampoule half an hour before being taken to the theater.

For the operation the patient should be given the half sitting posture, if possible, and the injured side should be dependent. If this position is not possible, the lung should be grasped with forceps of the Collin pattern immediately upon opening the thorax so as to correct displacement of the mediastinum.

The skin is prepared with picric acid. Paravertebral anesthesia is administered for two or three spaces above and below the wound. A local infiltration around but at some distance from the wound is added. Novocain 0.5 o/o, potassium sulfate 0.25 o/o in normal saline is injected with Gray's syringe. Never should ether or chloroform be used in chest surgery. Resection of the fourth rib from the mid-clavicular to the posterior axillary line furnishes the easiest access to the thoracic cavity. Operation should be as rapid as possible. It is important to avoid strong retraction on the chest wall, whereby shock is increased and the mediastinum disturbed.

A missile should never be left in the liver. The track in the liver should be thoroughly cleansed. After this operation the diaphragm should be closed with mattress sutures of catgut on a blunt needle. The authors do not consider it necessary, however, to suture the diaphragm to the wall in case it is stripped from its parietal attachment. It is wiser to deal with wounds of the abdomen after closing the chest. The utmost care should be exercised to cleanse the wound of all debris before closing. The chest should always be closed unless there is extensive gas infection of the lung tissue itself adherent to the wall. The chest must be hermetically closed with the first layer of muscle and close approximation of skin edges is essential. No fluid should be allowed to collect in the pleural cavity after the operation.

The use of 7 in. adhesive plaster to secure the dressing is helpful, in that it tends also to relieve the strain on the sutures and leaves the uninjured side free for expansion.

After the operation the patient should be given a semi-recumbent position inclined towards the injured side.

A " two step " operation is indicated when extensive trauma makes it necessary to treat thoroughly both orifices of a through and through wound, or when there is laceration of the diaphragm on both sides. Resection and drainage are rarely necessary and should not be resorted to unless severe constitutional symptoms of sepsis are present.

If there is undue restlessness after the operation, omnopon in small doses is freely administered. Every possible means of counteracting acidosis is employed.

Broncho-pneumonia is a complication greatly to be feared. If a case shows signs of this complication on the uninjured side early operation must not be lightly undertaken, but, if undertaken, only local anesthesia should be used.

